# Coffee Consumption Decreases Risks for Hepatic Fibrosis and Cirrhosis: A Meta-Analysis

**DOI:** 10.1371/journal.pone.0142457

**Published:** 2015-11-10

**Authors:** Fen Liu, Xiwei Wang, Gang Wu, Ling Chen, Peng Hu, Hong Ren, Huaidong Hu

**Affiliations:** 1 Department of Clinical Nutrition, the Second Affiliated Hospital of Chongqing Medical University, Chongqing, PR, China; 2 Department of Infectious Diseases, Institute for Viral Hepatitis, Key Laboratory of Molecular Biology for Infectious Diseases, The Second Affiliated Hospital of Chongqing Medical University, Chongqing, PR, China; 3 Department of Infectious Diseases, The Affiliated Hospital of LuZhou Medical College, LuZhou, China; Harvard Medical School, UNITED STATES

## Abstract

**Background and Aim:**

Previous studies have demonstrated that coffee consumption may be inversely correlated with hepatic fibrosis and cirrhosis. However, the reported results have been inconsistent. To summarize previous evidences quantitatively, a meta-analysis was performed.

**Methods:**

The Medline, Web of Science, and Embase databases (from inception to June 2015) were searched to identify relevant trials that evaluated the effects of coffee consumption on hepatic fibrosis or cirrhosis. Odds ratios (ORs) of advanced hepatic fibrosis or cirrhosis for low or moderate, high, and any coffee consumption versus no consumption were pooled. Two cups per day was used as the cut-off level between low or moderate and high consumption.

**Results:**

Sixteen studies were included, involving 3034 coffee consumers and 132076 people who do not consume coffee. The pooled results of the meta-analysis indicated that coffee consumers were less likely to develop cirrhosis compared with those who do not consume coffee, with a summary OR of 0.61 (95%CI: 0.45–0.84). For low or moderate coffee consumption versus no consumption, the pooled OR of hepatic cirrhosis was 0.66 (95%CI: 0.47–0.92). High coffee consumption could also significantly reduce the risk for hepatic cirrhosis when compared with no coffee consumption (OR = 0.53, 95%CI: 0.42–0.68). The effect of coffee consumption on hepatic fibrosis was summarized as well. The pooled OR of advanced hepatic fibrosis for coffee consumption versus no consumption was 0.73 (95%CI: 0.58–0.92). The protective effect of coffee on hepatic fibrosis and cirrhosis was also identified in subgroup meta-analyses of patients with alcoholic liver disease and chronic hepatitis C virus (HCV) infection.

**Conclusion:**

Coffee consumption can significantly reduce the risk for hepatic fibrosis and cirrhosis.

## Introduction

Hepatic fibrosis and cirrhosis are the major causes of mortality and morbidity in patients with chronic liver disease (CLD), and are a global health burden [1, 2]. Persistent liver injury caused by CLD can induce scar formation and healing, which consequently lead to fibrosis and cirrhosis [3, 4]. The 5-year cumulative incidence of decompensation in cirrhotic patients is approximately 20%. Those patients with decompensated cirrhosis only have a 5-year survival rate of 14–35% [5]. Worse still, the risk for hepatocellular carcinoma greatly increases after the development of fibrosis and cirrhosis.

Controlling CLD is the most important approach to prevent hepatic fibrosis. However, dietary factors may also have protective effects on liver diseases [6, 7]. Coffee intake has been found to be inversely correlated with serum levels of alanine aminotransferase (ALT) and gamma-glutamyltransferase (GGT), both of which are markers of liver injury and indicators of hepatic fibrosis [8–10]. Moreover, coffee is able to prevent hepatocellular carcinoma development that is mainly caused by hepatic fibrosis and cirrhosis [11, 12]. Recently, some studies have evaluated the association of coffee consumption with hepatic fibrosis and cirrhosis [13–15]. However, the reported results have been inconsistent, and no comprehensive meta-analyses have been conducted previously.

Meta-analysis is an important method to summarize the results from multiple studies quantitatively as well as to generate a final conclusion, especially when the results are inconsistent [16]. The goal of the present meta-analysis was to summarize the effects of low or moderate, high, and any coffee consumption on hepatic fibrosis and cirrhosis, compared with no consumption, in a quantitative manner.

## Methods

### Search strategy and study selection

A literature search was performed by two reviewers (FL and WXW). Medline, Web of Science, and Embase were searched from inception to June 2015, using the following combination of text words and Mesh terms: “beverage” or “caffeine” or “coffee”, “risk”, combined with “liver” or “hepatic cirrhosis” or “liver cirrhosis” or “hepatic fibrosis” or “liver fibrosis”. All epidemiological trials that evaluated the effects of coffee consumption on hepatic fibrosis or cirrhosis were included. Relevant reference lists were also screened to identify additional potential studies. Furthermore, manual searches and correspondence with authors were performed when necessary.

Studies were included if they met the following criteria: (a) cohort or case-control study, (b) coffee consumption was the exposure of interest, (c) focused on primary outcomes of advanced hepatic fibrosis or cirrhosis diagnosed according to the guidelines of the American Association for the Study of Liver Diseases (d) reported odds ratios (OR) or relative risk with relevant 95% confidence intervals (CIs) or data to calculate them. Studies that did not provide adequate information of primary outcomes or were not published in English were excluded. When multiple publications were identified from the same population, only the most complete one was selected. The Newcastle-Ottawa Scale was used to assess the quality of the included studies. Studies with total scores of more than six were considered to be of high quality [17].

### Data extraction

Each included study was abstracted in duplicate and evaluated independently by FL and WXW using a data collection form. The following data were extracted: (a) study characteristics (author, year of publication, sample size, and study design), (b) details of coffee consumption, (c) types of primary outcome (advanced hepatic fibrosis or cirrhosis) and (d) measurement of association (OR or relative risk) or data to calculate them, and adjusted covariates in the analysis. Estimates adjusted for potential covariates were utilized whenever possible.

Any conflicting opinions on study selection or data extraction were resolved by consensus of the investigators. The corresponding author (HDH) also provided arbitration when necessary.

### Statistical analysis

The effects of coffee consumption on hepatic fibrosis or cirrhosis were assessed by ORs combined with the corresponding 95%CIs. Summary ORs for low or moderate, high, and any coffee consumption versus no consumption were calculated. Due to the variances of coffee consumption among included studies, a coffee intake ≥ 2 cups per day was defined as high consumption. Coffee intake < 2 cups per day was defined as low or moderate consumption. Summary estimates were calculated by the fixed-effects model [18] or the random-effects model [19]. If no heterogeneity is identified among the studies, these two models will generate identical results. However, when heterogeneity is found, the 95%CI of the summary estimate calculated by the random-effects model will be wider than that calculated using the fixed-effects model [20]. The statistical significance claims of the random-effects model will also be more conservative. The heterogeneity among the included studies was evaluated by Cochran’s Q test and the I^2^ statistic. If the Q-test gave a p value < 0.1 to indicate significant heterogeneity [21], the summary estimates would be pooled using a random-effects model. The study weights were adjusted based on the extent of variation among the included studies when a random-effects model was used. Summary estimates were calculated as weighted averages of the estimates in the individual studies [20].

One-way sensitivity analysis was conducted to estimate the credibility of the pooled results. Galbraith plot was used to search for a potential source of heterogeneity. The outliers identified by the Galbraith plot were considered as the sources of heterogeneity [22]. Funnel plots were calculated to assess publication bias, together with Egger's regression asymmetry test, and Begg’s rank correlation test [23, 24]. All statistical analyses were performed by Stata (version 12.0). All p values were two tailed. With the exception of Cochran’s Q-test, all tests with a p value < 0.05 were considered to be statistically significant.

## Results

**Fig 1:**
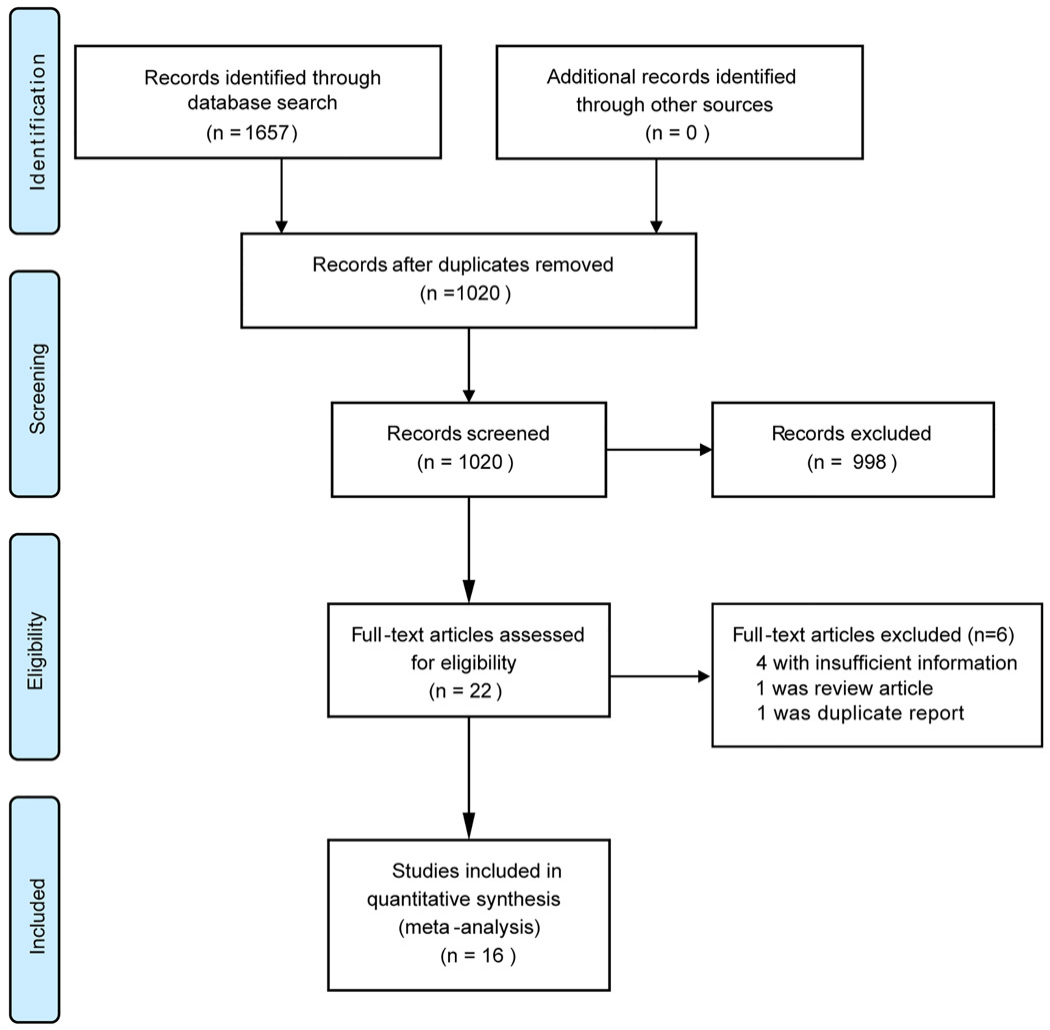
Study selection procedure.

Through database searches, 1657 potential citations were identified. After detailed evaluation and removal of duplicates, 16 studies were finally selected, including 7 case-control studies [14, 25–30] and 9 cohort studies [13, 15, 31–37] (Fig 1). In the case-control studies, previous coffee consumption of the participants was determined. In the cohort studies, coffee consumption at the study baseline was determined. Among the included studies, eight measured the associations of coffee consumption with cirrhosis development [14, 25–29, 31, 32], seven measured the associations of coffee consumption with hepatic fibrosis [13, 15, 30, 33, 34, 36, 37], and one reported on both the associations of coffee consumption with advanced hepatic fibrosis and cirrhosis [35]. Finally, 3034 coffee consumers and 132076 people who do not consume coffee were included. The study characteristics are shown in Table 1. Most of the included trials were of good quality.

**Table 1:** Study characteristics.

Study	Year	Country	Study design	No. of cases	No. of controls/cohort size	Outcome	Adjustments	Quality score*
Corrao	1994	Italy	case-control	115	167	cirrhosis	Smoking, alcohol consumption	8
Corrao	2001	Italy	case-control	274	458	cirrhosis	Education, age, HBsAg, alcohol consumption, HCV, intake of energy, carbohydrates, lipids, and proteins	8
GALLUS	2002	Italy	case-control	101	1538	cirrhosis	Age, sex, education, area of residence, year of interview, body mass index, diabetes, history of hepatitis, alcohol consumption, tobacco	6
Klatsky	2006	USA	cohort	330	125580	cirrhosis	Sex, race or ethnicity, smoking, alcohol consumption, education, body mass index	8
Freedman	2009	USA	cohort	331	776	cirrhosis	Age, body mass index, education, ethnicity, sex, baseline Ishak fibrosis score, total energy intake, lifetime alcohol consumption	9
Stroffolini	2010	Italy	case-control	136	613	cirrhosis	Alcohol consumption	7
Modi	2010	USA	cohort	54	177	fibrosis	Age, sex, race, body mass index, alcohol consumption	8
Ong	2011	Hong Kong	cohort	216	1045	fibrosis	Age, body mass index, alcohol consumption	7
Costentin	2011	France	cohort	55	238	fibrosis	Not specified	8
Anty	2012	France	cohort	68	195	fibrosis	AST, homeostatic model assessment-insulin resistance, metabolic syndrome, presence of nonalcoholic steatohepatitis	7
Walton	2013	UK	case-control	95	191	cirrhosis	Age, alcohol consumption	6
Triantos	2013	UK	case-control	240	391	cirrhosis	Age, gender, smoking, alcohol consumption	6
Machado	2014	Brazil	cohort	64	136	fibrosis	Not specified	7
El-Serag	2014	USA	cohort	355	597	fibrosis	Demographic, clinical, and other dietary variables	6
Bambha	2014	USA	cohort	258	782	both	Age, smoking, diabetes	6
Khalaf	2015	USA	case-control	342	568	fibrosis	Age, alcohol use, body mass index, metabolic syndrome	8

*: Study quality was assessed using the Newcastle-Ottawa Scale (score of 0–9). AST, aspartate transaminase; HBsAg, hepatitis B surface antigen; HCV, hepatitis C virus.

**Fig 2:**
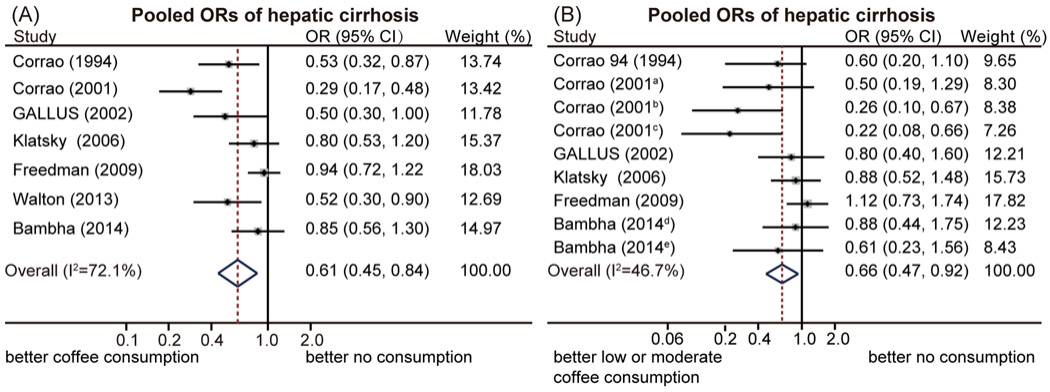
Pooled odds ratios (ORs) of hepatic cirrhosis. (A) Coffee consumption could significantly reduce the risk for hepatic cirrhosis, compared with no consumption. (B) Low or moderate coffee consumption could significantly reduce the risk for hepatic cirrhosis, compared with no consumption. Note: coffee consumption a: 0–100 mg/day; b: 101–200 mg/day; c: 201–300 mg/day; d: <1 cup/day; e: 1–2 cups/day.

Seven included studies reported the association of coffee consumption with hepatic cirrhosis, compared with no coffee consumption [14, 25, 26, 29, 31, 32, 35]. All of the studies identified that coffee intake could reduce the risk for cirrhosis, yet only three of them had results that reached statistical significance [14, 25, 29]. Significant heterogeneity among the studies was found (I^2^ = 72.1%; Q-test, p < 0.01). The pooled results of the meta-analysis indicated that coffee consumers were less likely to develop cirrhosis compared with those who do not consume coffee, with a summary OR of 0.61 (95%CI: 0.45–0.84, random-effects model, Fig 2A). The Galbraith plot found that a study from the USA showing no significant association [32] and a study from Italy reporting a much stronger association [14] mainly contributed to the heterogeneity (S1 Fig). A sensitivity analysis indicated that no single study significantly influenced the pooled estimate (S2 Fig). After removing the two studies identified as outliers in the Galbraith plot, heterogeneity was found to be significantly reduced, yet the pooled estimates were still stable (OR = 0.66, 95%CI: 0.52–0.83; Table 2). Subgroup analysis was performed according to the study quality. The pooled estimates were similar between low- and high-quality studies, with OR values of 0.64 (95%CI: 0.44–0.91) and 0.60 (95%CI: 0.36–0.99), respectively.

**Table 2:** Sensitivity analysis for meta-analysis of coffee consumption vs no consumption on cirrhosis development.

Meta-analysis	Omitted Study	No. of Included Study	OR	95% CI	P-het*	I^2^
Overall	0	7	0.61 (0.45–0.84)	0.447–0.841	p<0.01	72.10%
Sensitivity analysis 1	Freedman	6	0.56 (0.40–0.78)	0.403–0.777	0.02	62.80%
Sensitivity analysis 2	Corrao 2001	6	0.72 (0.57–0.90)	0.569–0.904	0.125	42.00%
Sensitivity analysis 3	Corrao 2001 and Freedman	5	0.66 (0.52–0.83)	0.524–0.828	0.34	11.50%

* P value of heterogenity.

**Fig 3:**
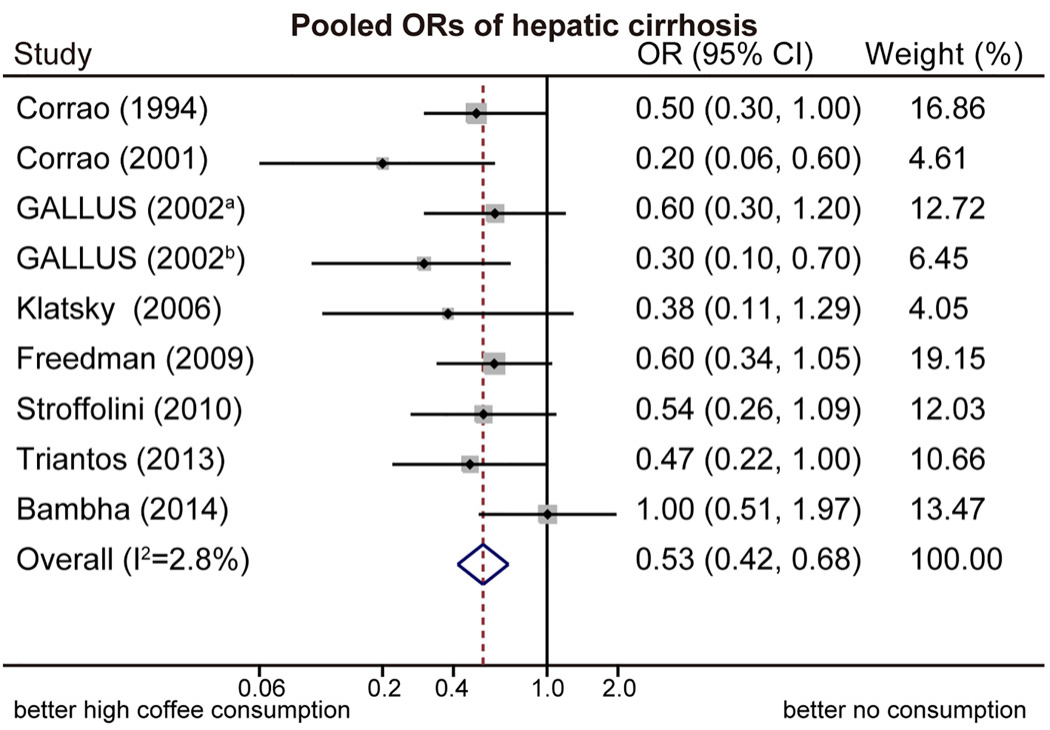
Pooled odds ratios (ORs) of hepatic cirrhosis. High coffee consumption significantly reduced the risk for hepatic cirrhosis, compared with no consumption. Note: a: 2 cups/day; b: ≥ 3 cups/day.

Nine subgroups in six included studies [14, 25, 26, 31, 32, 35] were reported about the associations of low or moderate coffee consumption with cirrhosis, compared with no coffee consumption. The pooled OR was 0.66 (95%CI: 0.47–0.92, random-effects model, Fig 2B), with significant heterogeneity identified among the included studies (I^2^ = 46.7%; Q-test, p = 0.059). Study-specific and overall estimates of cirrhosis for high coffee consumption compared to no consumption is shown in Fig 3. The pooled OR was 0.53 (95%CI: 0.42–0.68, fixed-effects model), based on nine subgroups in eight included studies [14, 25–28, 31, 32, 35]. No significant heterogeneity was found (I^2^ = 2.8%; Q-test, p = 0.441).

**Fig 4:**
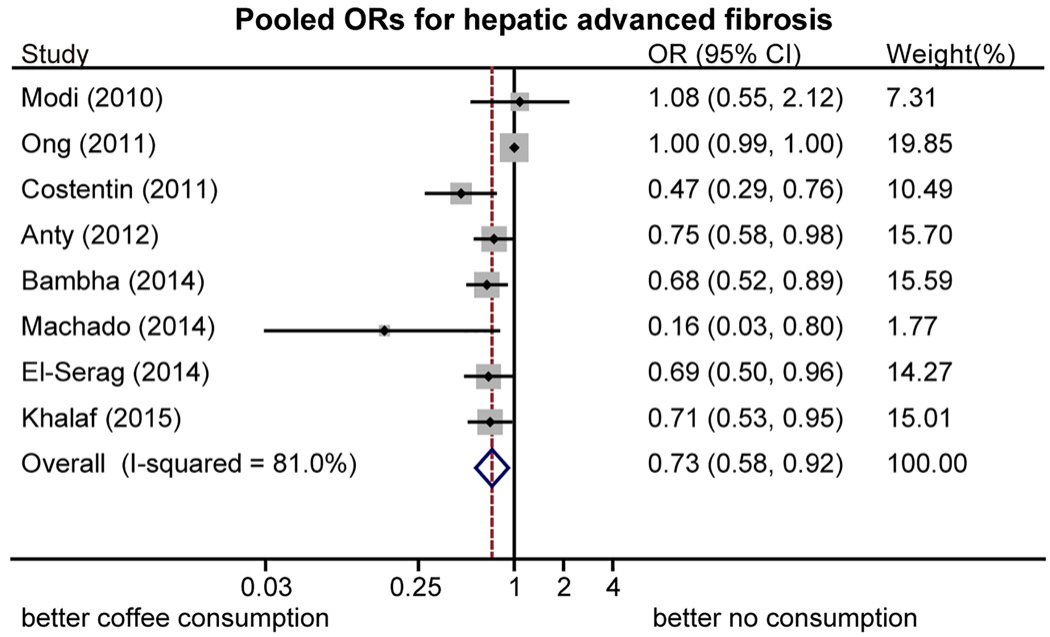
Pooled odds ratios (ORs) of advanced hepatic fibrosis. Coffee consumption significantly reduced the risk for advanced hepatic fibrosis, compared with no consumption.

Estimated ORs of advanced hepatic fibrosis for coffee consumption compared with no consumption are shown in Fig 4. The pooled results of eight studies [13, 15, 30, 33–37] showed that coffee consumers were less likely to develop fibrosis, compared with those who do not consume coffee (OR = 0.73, 95%CI: 0.58–0.92, random-effects model). Significant heterogeneity was observed among these studies (I^2^ = 80.9%, p < 0.01). A sensitivity analysis showed the robustness of the pooled results and identified a cohort study from Hong Kong [34] as the major source of heterogeneity because no significant association with a high weight percent was found.

The study-specific ORs and relevant CIs of cirrhosis or advanced hepatic fibrosis for coffee consumption compared with no consumption are presented in Table 3, stratified by the presence of hepatitis and alcohol drinking. Five studies [14, 25–27, 31] compared the effects of coffee consumption with no coffee consumption on cirrhosis development in alcohol drinking population, which rendered a summary OR of 0.49 (95%CI: 0.36–0.67; I^2^ = 43.2%, Q-test, p = 0.038; random-effects model). Moreover, five studies [15, 30, 33, 36, 37] compared the effects of coffee consumption with no coffee consumption on advanced hepatic fibrosis in patients infected with hepatitis C virus (HCV). The pooled OR was 0.65 (95%CI: 0.49–0.87; I^2^ = 46.3%, Q-test, p = 0.061; random-effects model). These resulsts suggested that coffee consumption has hepatoprotective effecf on hepatic fibrosis and cirrhosis in these two population.

**Table 3:** Study-specific odds ratios (ORs) and pooled ORs, with 95%CIs, for coffee consumption and hepatic fibrosis/cirrhosis, classified by strata of chronic liver disease and alcohol consumption.

Study	Year	Strata of covariate		Coffee consumption	OR
**Hepatic cirrhosis**					
Corrao	1994	Alcohol	1–100 g/day	Consumption vs. no consumption	0.54 (0.26–1.13)
			≥ 100 g/day	Consumption vs. no consumption	0.58 (0.14–2.43)
Corrao	2001	Alcohol	1–25 g/day	≤ 1 vs. no consumption	0.69 (0.27–1.79)
				>1 vs. no consumption	0.52 (0.22–1.25)
			26–50 g/day	≤ 1 vs. no consumption	1.25 (0.45–3.49)
				>1 vs. no consumption	0.53 (0.21–1.35)
			≥ 51 g/day	≤ 1 vs. no consumption	0.22 (0.08–0.66)
				>1 vs. no consumption	0.2 (0.07–0.54)
GALLUS	2002	Alcohol	< 3 drinks/day	≥ 2 vs. <2 cups/day	0.2 (0.1–0.6)
			≥ 3 drinks/day	≥ 2 vs. <2 cups/day	0.8 (0.4–1.6)
Klatsky	2006	Alcohol	drinking	<1 vs. no or seldom consumption	0.7 (0.4–1.1)
				1–3 vs. no or seldom consumption	0.6 (0.4–0.8)
				≥ 4 vs. no or seldom consumption	0.2 (0.1–0.4)
Freedman	2009	HCV		0–1 vs. no consumption	1.12 (0.73–1.74)
				1–3 vs. no consumption	1.03 (0.68–1.54)
				≥ 3 vs. no consumption	0.6 (0.34–1.05)
Stroffolini	2010	Alcohol	1–3 drinks/day	>2 vs. 0–2 cups/day	0.36 (0.1–1.28)
			>3 drinks/day	>2 vs. 0–2 cups/day	0.62 (0.25–1.55)
Triantos	2013	CLD		>2 vs. no consumption	0.47 (0.22–1)
Walton	2013	CLD		Consumption vs. no consumption	0.52 (0.3–0.9)
Bambha	2014	NAFLD		<1 vs. no consumption	0.88 (0.44–1.75)
				1–2 vs. no consumption	0.61 (0.24–1.56)
				≥ 2 vs. no consumption	1 (0.51–1.97)
**Pooled estimate of alcohol drinking population**				Consumption vs. no consumption	0.49 (0.36–0.67)
**Hepatic fibrosis**					
Modi	2010	HCV		43–125 mg/day vs. seldom consumption	0.96 (0.27–3.4)
				125–345 mg/day vs. seldom consumption	1.63 (0.82–3.3)
				345–1028 mg/day vs. seldom consumption	0.51 (0.27–0.98)
Costentin	2011	HCV		1.5–3 vs. <1.5 cups/day	0.23 (0.08–0.63)
				3–5 vs. 1.5 cups/day	0.57 (0.26–1.27)
				> 5 vs. 1.5 cups/day	0.65 (0.29–1.44)
Ong	2011	HBV		Consumption vs. no consumption	1 (0.99–1)
Anty	2012	NAFLD		Consumption vs. no consumption	0.75 (0.58–0.98)
Machado	2014	HCV		Consumption vs. no consumption	0.16 (0.03–0.8)
El-Serag	2014	HCV		Consumption vs. no consumption	0.69 (0.5–0.96)
Khalaf	2015	HCV		Low consumption vs. no consumption	0.71 (0.53–0.95)
**Pooled estimate of HCV population**				Moderate/high vs. no/low consumption	0.65 (0.49–0.87)

CLD, chronic liver disease; HBV, hepatitis B virus; HCV, hepatitis C virus; NAFLD, nonalcoholic fatty liver disease.

## Discussion

Coffee consumption might reduce the risks for hepatic fibrosis and cirrhosis. However, previous studies have reported inconsistent results and failed to provide a conclusive recommendation [14, 25–29, 31, 32, 35]. In the present meta-analysis, the pooled estimates demonstrated significantly reduced risks for hepatic cirrhosis and advanced fibrosis in coffee consumers. More importantly, the included studies that reported hepatoprotective effects of coffee were mostly from Western countries where coffee consumption is frequent [13–15, 25–29, 31–33, 35, 36].

The mechanism of the protective effects of coffee consumption on hepatic fibrosis and cirrhosis remains to be elucidated. Several studies have identified preventive effects of coffee and caffeine extracts on hepatic fibrosis in standard rodent models. For example, Shim *et al*. have found that in an immortalized human hepatic stellate cell (HSC) line, caffeine can reduce the risk for hepatic fibrosis by downregulating the expression of α-smooth muscle actin and procollagen type Ic as well as inducing apoptosis of HSCs [38]. Decreased liver inflammation and fibrosis were also observed in thioacetamide-treated rats used in the study by Shim *et al*. Moreover, Moreno *et al*. have demonstrated that coffee consumption reduced collagen content and the levels of the profibrogenic cytokine transforming growth factor, which consequently prevented the development of hepatic cirrhosis in a CCl_4_-treated rat model [39]. Another potential mechanism is that coffee intake might significantly alleviate liver inflammation, since it has been shown to have a potent antioxidant capacity by increasing the plasma concentration of glutathione, thus preventing scar formation and promoting healing [40]. Moreover, coffee consumption might prevent liver damage by reducing systemic and liver oxidative stress as well as decreasing the concentrations of proinflammatory and inflammatory cytokines in the liver [41]. As HSCs are the major effector cells in the pathogenesis of hepatic fibrosis/cirrhosis, it seems that the suppression of HSC A 2a adenosine receptors by caffeine is the primary mechanism to prevent fibrosis/cirrhosis [42].

Coffee consumption has been found to be correlated with a reduction of ALT activity in a large population-based study of CLD [43]. Moreover, it also decreased GGT levels and protected liver cells from damage [10, 44]. As ALT and GGT activity are well-established risk indicators for hepatic fibrosis and cirrhosis [45, 46], the hepatoprotective effects of coffee on hepatic fibrosis and cirrhosis might partly be modulated by the reduction of ALT and GGT.

Hepatic fibrosis and cirrhosis caused by CLD are global health problems. Therefore, it is important to develop some simple approaches to prevent fibrosis and cirrhosis in patients with high risk. Through subgroup meta-analysis, we identified significant hepatoprotective effects of coffee consumption on hepatic fibrosis and cirrhosis in patients with alcoholic liver disease and chronic HCV infection, which mainly contribute to CLD in Western countries. Thus, coffee consumption seems to be an attractive lifestyle for CLD patients, as coffee intake is popular in those areas. In addition to its antifibrotic effect, coffee can reduce the risk for liver cancer [12, 47], which is the worst outcome for CLD patients.

As the present study is based on the evidences of observational studies, it is difficult to determine whether it is occasional for the inverse associations of coffee consumption with hepatic fibrosis and cirrhosi. Caffeine metabolism in CLD patients has been reported to be impaired due to liver injury [48]. Therefore, the inverse associations may partly be due to the reduction of coffee consumption caused by digestive tract discomfort. This could have rendered a spurious protective effect of coffee on hepatic fibrosis and cirrhosis. However, all the included studies used CLD patients as controls to eliminate such a possibility.

There are some limitations to the present study. First, most of the included studies were in Western countries. Thus, generalization of the current findings to other populations should be made with caution. In addition, analysis of publication bias could not be conducted due to limited studies in each pooled meta-analysis. As null results in small studies tend not to be published, the associations of coffee consumption with hepatic fibrosis and cirrhosis development may have been overestimated due to potential publication bias. Third, there was variance in consumption duration among the included studies, which might have been a potential source of heterogeneity. Therefore, the conclusion of the current study that coffee consumption can reduce the risk for hepatic fibrosis/cirrhosis might be underestimated because some differences among the study groups might not have been identified if the consumption duration was insufficient. Finally, the estimations of coffee consumption in the included trials were mainly obtained by self-reporting, which might have caused some bias. Nevertheless, this assessment has been previously shown to be valid and reproducible [49, 50].

In conclusion, the present meta-analysis suggests that coffee consumption can prevent the development of hepatic fibrosis and cirrhosis. However, further prospective studies are still needed to control the bias and confounding factors.

## Supporting Information

S1 Fig Galbraith plot for meta-analysis of coffee consumption versus no consumption in cirrhosis development. Two studies, as the outliers, were found to be the potential source of heterogeneity.
(TIF)

S2 Fig Sensitivity analysis of pooled estimates of liver cirrhosis for coffee consumption versus no consumption. No single study was found to significantly influence the pooled estimate.
(TIF)

S1 Table PRISMA checklist. (DOC)
