# Supplementary figures and images for: Coffee Consumption Decreases Risks for Hepatic Fibrosis and Cirrhosis: A Meta-Analysis
Source: PLoS One. 2015 Nov 10;10(11):e0142457. doi: 10.1371/journal.pone.0142457 (PMC4640566; doi:10.1371/journal.pone.0142457)

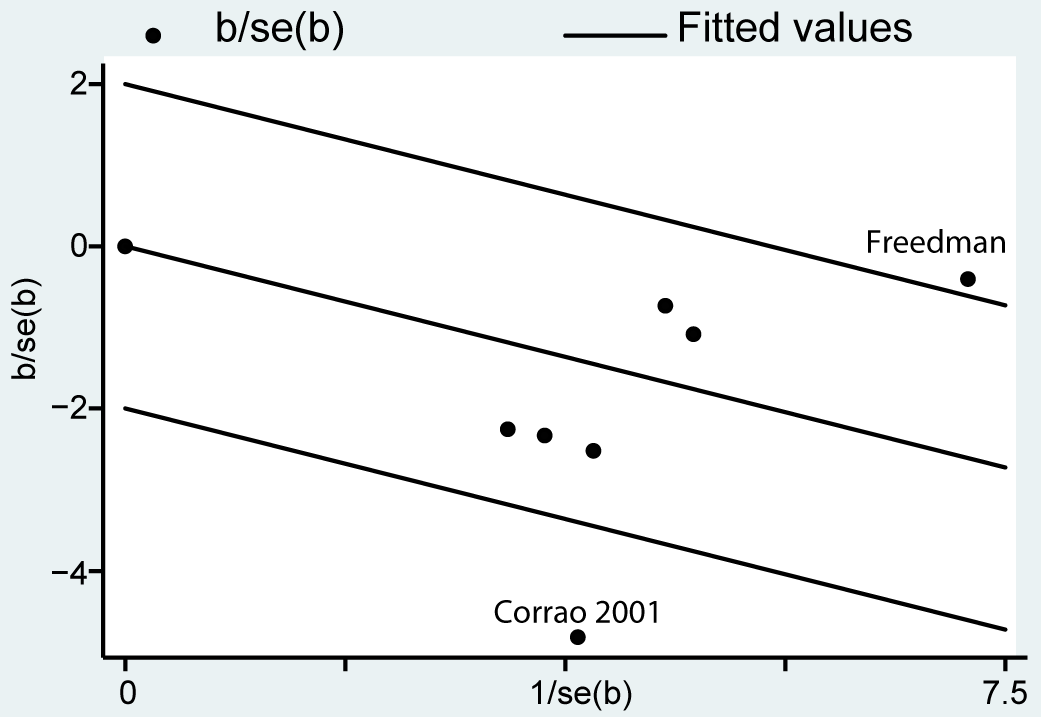

Supplement: S1 Fig — Two studies, as the outliers, were found to be the potential source of heterogeneity. (TIF) [file pone.0142457.s001.tif]

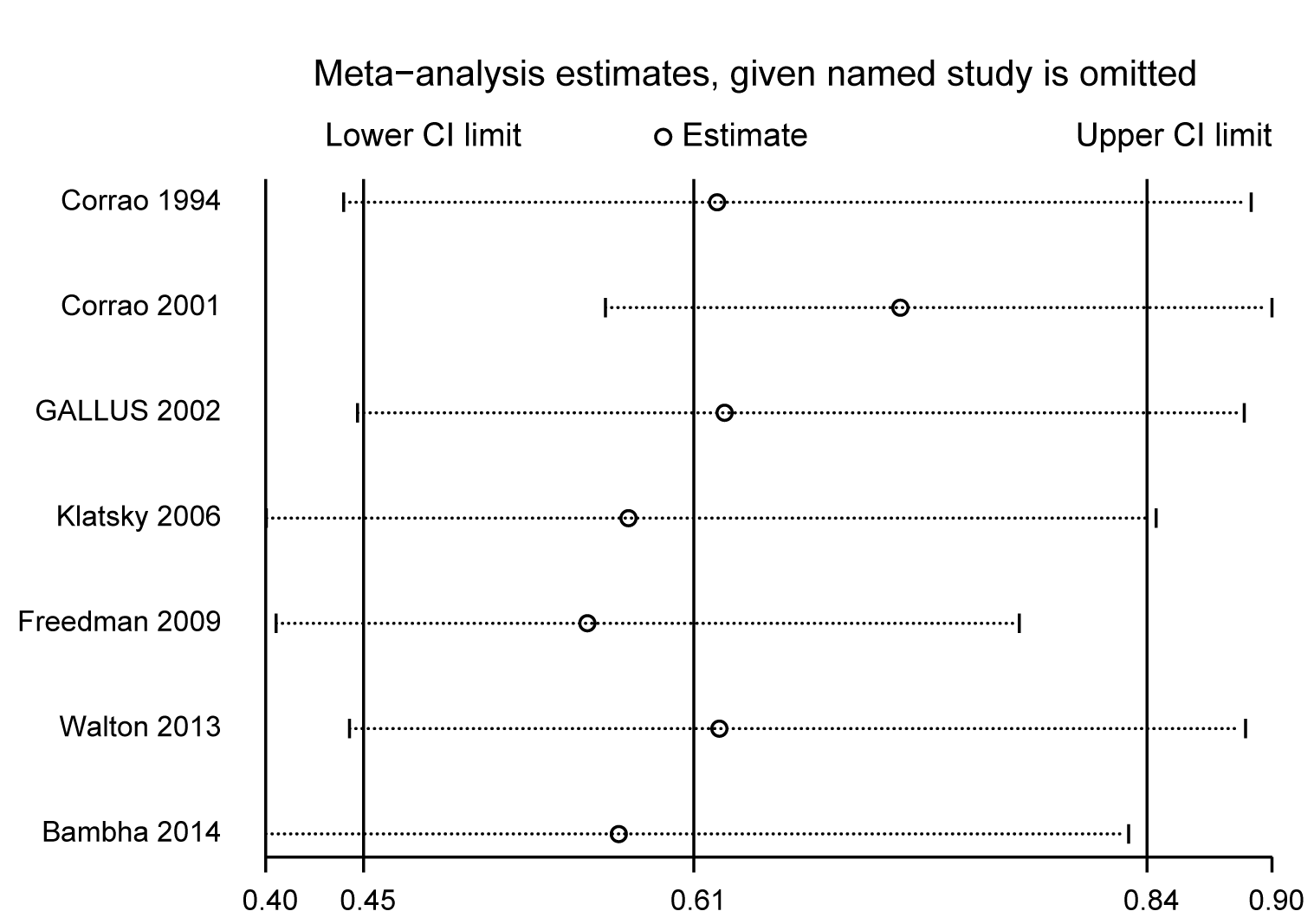

Supplement: S2 Fig — No single study was found to significantly influence the pooled estimate. (TIF) [file pone.0142457.s002.tif]
